# Poly[[aqua­[μ-1,4-bis­(3-pyridylmeth­yl)piperazine-κ^2^ *N*:*N*′](μ-isophthalato-κ^2^ *O* ^1^:*O* ^3^)copper(II)]

**DOI:** 10.1107/S160053681000632X

**Published:** 2010-02-20

**Authors:** Chaun M. Gandolfo, Robert L. LaDuca

**Affiliations:** a Lyman Briggs College, Department of Chemistry, Michigan State University, East Lansing, MI 48825 USA

## Abstract

In the title compound, [Cu(C_8_H_4_O_4_)(C_16_H_20_N_4_)(H_2_O)]_*n*_, square-pyramidally coordinated Cu^II^ ions are linked into [Cu(H_2_O)(isophthalate)]_*n*_ coordination polymer chains by isophthalate dianions. These chains are connected into undulating [Cu(H_2_O)(isophthalate)(3-bpmp)]_*n*_ [3-bmp is bis­(3-pyridylmeth­yl)piperazine] layers by 3-bpmp tethering ligands. The *pseudo* three-dimensional structure of the title compound is fostered by inter­layer O—H⋯O hydrogen bonding between the aqua ligands and unligated isophthalate O atoms. The selected crystal was non-merohedrally twinned. Only reflections from the major twin component were used in the solution and refinement.

## Related literature

For other divalent copper aromatic dicarboxyl­ate coordination polymers containing bis­(3-pyridylmeth­yl)piperazine, see: Johnston *et al.* (2008 ▶). For the synthesis of bis­(3-pyridyl­meth­yl)piperazine, see: Pocic *et al.* (2005 ▶). The twin law was determined using *CELLNOW* (Sheldrick, 2009 ▶).
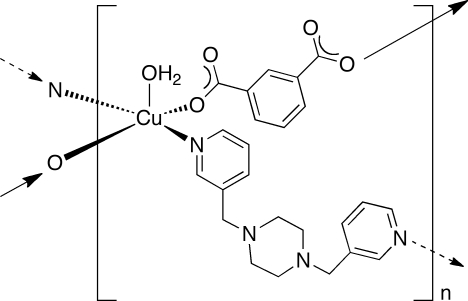


## Experimental

### 

#### Crystal data

[Cu(C_8_H_4_O_4_)(C_16_H_20_N_4_)(H_2_O)]
*M* *_r_* = 514.03
Triclinic, 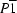

*a* = 6.9122 (4) Å
*b* = 10.0328 (5) Å
*c* = 16.7456 (9) Å
α = 86.822 (1)°
β = 84.210 (1)°
γ = 80.771 (1)°
*V* = 1139.49 (11) Å^3^
*Z* = 2
Mo *K*α radiation
μ = 1.00 mm^−1^
*T* = 173 K
0.34 × 0.18 × 0.17 mm

#### Data collection

Bruker APEXII diffractometer
Absorption correction: multi-scan (*TWINABS*; Sheldrick, 2003 ▶) *T*_min_ = 0.729, *T*_max_ = 0.850
31818 measured reflections
4141 independent reflections
3681 reflections with *I* > 2σ(*I*)
*R*_int_ = 0.077

#### Refinement

*R*[*F* ^2^ > 2σ(*F* ^2^)] = 0.043
*wR*(*F* ^2^) = 0.117
*S* = 1.16
4141 reflections
313 parameters
3 restraints
H atoms treated by a mixture of independent and constrained refinement
Δρ_max_ = 0.44 e Å^−3^
Δρ_min_ = −0.56 e Å^−3^

### 

Data collection: *APEX2* (Bruker, 2006 ▶); cell refinement: *SAINT* (Bruker, 2006 ▶); data reduction: *SAINT*; program(s) used to solve structure: *SHELXS97* (Sheldrick, 2008 ▶); program(s) used to refine structure: *SHELXL97* (Sheldrick, 2008 ▶); molecular graphics: *Crystal Maker* (Palmer, 2007 ▶); software used to prepare material for publication: *SHELXL97*.

## Supplementary Material

Crystal structure: contains datablocks I, pub. DOI: 10.1107/S160053681000632X/ng2734sup1.cif

Structure factors: contains datablocks I. DOI: 10.1107/S160053681000632X/ng2734Isup2.hkl

Additional supplementary materials:  crystallographic information; 3D view; checkCIF report

## Figures and Tables

**Table 1:** Hydrogen-bond geometry (Å, °)

*D*—H⋯*A*	*D*—H	H⋯*A*	*D*⋯*A*	*D*—H⋯*A*
O5—H5*A*⋯O1^i^	0.82 (2)	1.96 (2)	2.778 (3)	174 (3)
O5—H5*B*⋯O3^ii^	0.83 (2)	2.02 (2)	2.805 (3)	158 (3)

Symmetry codes: (i) 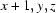; (ii) 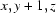.

**Fig. 1.**
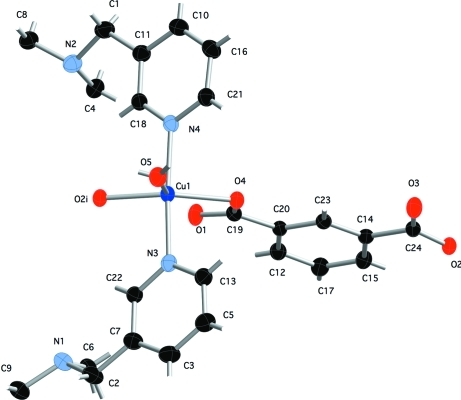
The asymmetric unit of the title compound, showing 50% probability ellipsoids and atom numbering scheme. Hydrogen atom positions are shown as grey sticks. Color codes: dark blue Cu, light blue N, red O, black C. Symmetry codes: (i) x, y, z.

**Fig. 2.**
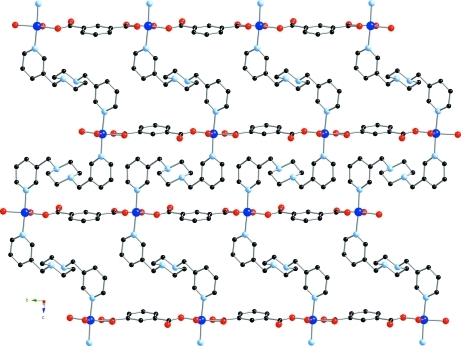
Face-on view of the coordination polymer layer motif in the title compound.

**Fig. 3.**
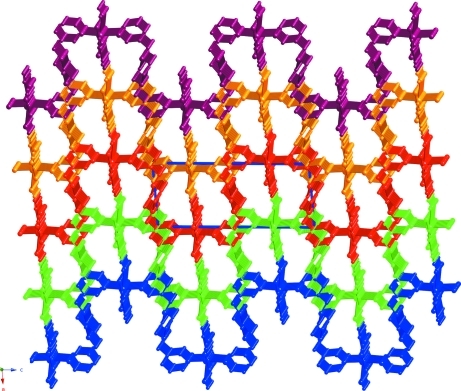
Stacking diagram for the title compound, viewed down the b axis.

## supplementary crystallographic information

### Comment

The title compound (I) was prepared by the hydrothermal reaction of copper nitrate, isophthalic acid and bis(3-pyridylmethyl)piperazine (3-bpmp). Its asymmetric unit (Fig. 1) contains a divalent copper atom, an aqua ligand, a fully deprotonated isophthalate ligand, and halves of two crystallographically independent 3-bpmp ligands whose central piperazinyl rings contain crystallographic inversion centers. In contrast to the previously reported phase {[Cu(H_2_O)(isophthalate)(3-bpmp)]^.^2H_2_O}_n_ (Johnston, *et al.*, 2008) no water molecules of crystallization are present in the title compound.

The basal plane of the distorted square pyramidal {CuO_3_N_2_} coordination sphere contains two *trans* pyridyl N atom donors from crystallographically distinct 3-bpmp ligands and two *trans* O atom donors from different isophthalate ligands. The apical position is occupied by the aqua ligand.

Isophthalate ligands in a bis(monodentate) bridging mode link the Cu^II^ ions into one-dimensional [Cu(H_2_O)(isophthalate)]_n_ coordination polymer chains arranged along the *b* crystal direction. The Cu···Cu distance through the isophthalate ligands measures 10.0328 (5) Å, defining the *b* lattice parameter. In turn, these chains are connected into undulating [Cu(H_2_O)(isophthalate)(3-bpmp)]_n_ coordination polymer layers by 3-bpmp tethering ligands (Fig. 2). These layers are arranged parallel to the *bc* crystal planes. The crystallographically distinct 3-bpmp ligands promote two different through-ligand Cu···Cu contact distances, 12.394 (6) and 12.830 (6) Å. The "wavelength" of the undulations in the layer motifs is 16.7456 (9) Å, which defines the *c* lattice parameter. Intralayer hydrogen bonding between the aqua ligands and unligated isophthalate O atoms provides additional stabilization of the layer motifs.

Adjacent [Cu(H_2_O)(isophthalate)(3-bpmp)]_n_ layers stack in an *AAA* pattern along the *a* crystal direction. Interlayer hydrogen bonding between the aqua ligands and unligated isophthalate O atoms provides the supramolecular interactions necessary to generate the *pseudo* three-dimensional structure of the title compound.

### Experimental

All starting materials were obtained commercially, except for 3-bpmp, which was prepared according to a literature procedure (Pocic, *et al.*, 2005). A mixture of Cu(NO_3_)_2_^.^2.5H_2_O (72 mg, 0.30 mmol), isophthalic acid (49 mg, 0.30 mmol), and 3-bpmp (79 mg, 0.30 mmol) and 10.0 g water (550 mmol) was placed into a 15 ml borosilicate glass vial, which was then sealed and heated under autogenous pressure at 363 K for 120 h. Blue-green blocks of the title compound were obtained along with a green polycrystalline material.

### Refinement

All H atoms bound to C atoms were placed in calculated positions, with C—H = 0.95 - 0.99 Å, and refined in riding mode with *U*_iso_ = 1.2*U*_eq_(C). The H atoms bound to the aqua ligand O atom was found in a difference Fourier map, restrained with O—H = 0.85 Å, and refined with *U*_iso_ =1.2*U*_eq_(O).

### Crystal data

**Table:**

[Cu(C_8_H_4_O_4_)(C_16_H_20_N_4_)(H_2_O)]	*Z* = 2
*M**_r_* = 514.03	*F*(000) = 534
Triclinic, *P*1	*D*_x_ = 1.498 Mg m^−^^3^
*a* = 6.9122 (4) Å	Mo *K*α radiation, λ = 0.71073 Å
*b* = 10.0328 (5) Å	Cell parameters from 4141 reflections
*c* = 16.7456 (9) Å	θ = 1.2–25.3°
α = 86.822 (1)°	µ = 1.00 mm^−^^1^
β = 84.210 (1)°	*T* = 173 K
γ = 80.771 (1)°	Block, blue-green
*V* = 1139.49 (11) Å^3^	0.34 × 0.18 × 0.17 mm

### Data collection

**Table:**

Bruker APEXII diffractometer	4141 independent reflections
Radiation source: fine-focus sealed tube	3681 reflections with *I* > 2σ(*I*)
graphite	*R*_int_ = 0.077
ω–φ scans	θ_max_ = 25.3°, θ_min_ = 1.2°
Absorption correction: multi-scan (TWINABS; Sheldrick, 2003)	*h* = −8→8
*T*_min_ = 0.729, *T*_max_ = 0.850	*k* = −11→12
31818 measured reflections	*l* = 0→20

### Refinement

**Table:**

Refinement on *F*^2^	Primary atom site location: structure-invariant direct methods
Least-squares matrix: full	Secondary atom site location: difference Fourier map
*R*[*F*^2^ > 2σ(*F*^2^)] = 0.043	Hydrogen site location: inferred from neighbouring sites
*wR*(*F*^2^) = 0.117	H atoms treated by a mixture of independent and constrained refinement
*S* = 1.16	*w* = 1/[σ^2^(*F*_o_^2^) + (0.038*P*)^2^ + 0.3004*P*] where *P* = (*F*_o_^2^ + 2*F*_c_^2^)/3
4141 reflections	(Δ/σ)_max_ < 0.001
313 parameters	Δρ_max_ = 0.44 e Å^−^^3^
3 restraints	Δρ_min_ = −0.56 e Å^−^^3^

### Special details

**Table:**

Experimental. The selected crystal was non-merohedrally twinned. The twin law was determined using CELLNOW (Sheldrick, 2009). Only reflections from the major twin component were used in the solution and refinement.
Geometry. All esds (except the esd in the dihedral angle between two l.s. planes) are estimated using the full covariance matrix. The cell esds are taken into account individually in the estimation of esds in distances, angles and torsion angles; correlations between esds in cell parameters are only used when they are defined by crystal symmetry. An approximate (isotropic) treatment of cell esds is used for estimating esds involving l.s. planes.
Refinement. Refinement of *F*^2^ against ALL reflections. The weighted *R*-factor wR and goodness of fit *S* are based on *F*^2^, conventional *R*-factors *R* are based on *F*, with *F* set to zero for negative *F*^2^. The threshold expression of *F*^2^ > σ(*F*^2^) is used only for calculating *R*-factors(gt) etc. and is not relevant to the choice of reflections for refinement. *R*-factors based on *F*^2^ are statistically about twice as large as those based on *F*, and *R*- factors based on ALL data will be even larger.

### Fractional atomic coordinates and isotropic or equivalent isotropic displacement parameters (Å^2^)

**Table:**

	*x*	*y*	*z*	*U*_iso_*/*U*_eq_	
Cu1	0.06345 (5)	0.16501 (3)	0.247474 (17)	0.03608 (14)	
O1	−0.2554 (3)	0.07048 (19)	0.26910 (13)	0.0497 (5)	
O2	−0.0541 (3)	−0.64683 (17)	0.24333 (11)	0.0407 (4)	
O3	0.1918 (3)	−0.53485 (19)	0.26226 (12)	0.0481 (5)	
O4	0.0528 (3)	−0.03315 (17)	0.25491 (10)	0.0378 (4)	
O5	0.3884 (3)	0.2070 (2)	0.22304 (13)	0.0468 (5)	
H5A	0.489 (4)	0.165 (3)	0.2398 (18)	0.056*	
H5B	0.362 (5)	0.2875 (19)	0.2338 (19)	0.056*	
N1	−0.2981 (4)	0.4364 (2)	−0.00903 (14)	0.0450 (6)	
N2	−0.3058 (4)	0.4306 (2)	0.50868 (14)	0.0459 (6)	
N3	0.0735 (3)	0.1569 (2)	0.12699 (13)	0.0369 (5)	
N4	0.0851 (3)	0.1613 (2)	0.36631 (13)	0.0364 (5)	
C1	−0.1526 (5)	0.3472 (3)	0.55036 (17)	0.0522 (7)	
H1A	−0.2147	0.2988	0.5967	0.063*	
H1B	−0.0697	0.4063	0.5715	0.063*	
C2	−0.1293 (5)	0.3582 (3)	−0.05256 (17)	0.0543 (8)	
H2A	−0.0398	0.4206	−0.0763	0.065*	
H2B	−0.1754	0.3150	−0.0973	0.065*	
C3	0.1232 (4)	0.1509 (3)	−0.03844 (18)	0.0516 (7)	
H3	0.1412	0.1486	−0.0954	0.062*	
C4	−0.4673 (4)	0.3578 (3)	0.49701 (18)	0.0492 (7)	
H4A	−0.5319	0.3322	0.5498	0.059*	
H4B	−0.4150	0.2741	0.4683	0.059*	
C5	0.2344 (4)	0.0554 (3)	0.00767 (18)	0.0505 (7)	
H5	0.3287	−0.0133	−0.0168	0.061*	
C6	−0.4586 (4)	0.3589 (3)	0.01158 (18)	0.0486 (7)	
H6A	−0.4119	0.2774	0.0446	0.058*	
H6B	−0.5022	0.3290	−0.0381	0.058*	
C7	−0.0140 (4)	0.2497 (3)	−0.00209 (17)	0.0447 (7)	
C8	−0.3847 (4)	0.5539 (3)	0.55103 (17)	0.0471 (7)	
H8A	−0.2761	0.6037	0.5592	0.056*	
H8B	−0.4486	0.5304	0.6044	0.056*	
C9	−0.3711 (4)	0.5567 (3)	−0.05738 (17)	0.0490 (7)	
H9A	−0.4137	0.5290	−0.1078	0.059*	
H9B	−0.2640	0.6109	−0.0716	0.059*	
C10	0.1134 (5)	0.1464 (3)	0.53032 (18)	0.0484 (7)	
H10	0.1234	0.1402	0.5866	0.058*	
C11	−0.0233 (4)	0.2453 (3)	0.49772 (16)	0.0429 (6)	
C12	−0.3987 (4)	−0.1727 (3)	0.24824 (15)	0.0399 (6)	
H12	−0.4912	−0.0917	0.2477	0.048*	
C13	0.2053 (4)	0.0623 (3)	0.09015 (17)	0.0444 (6)	
H13	0.2822	−0.0029	0.1221	0.053*	
C14	−0.1298 (4)	−0.4115 (3)	0.25201 (14)	0.0351 (6)	
C15	−0.3263 (4)	−0.4149 (3)	0.24274 (17)	0.0422 (6)	
H15	−0.3696	−0.4990	0.2377	0.051*	
C16	0.2351 (4)	0.0567 (3)	0.48069 (18)	0.0478 (7)	
H16	0.3297	−0.0113	0.5024	0.057*	
C17	−0.4595 (4)	−0.2960 (3)	0.24079 (17)	0.0438 (6)	
H17	−0.5936	−0.2992	0.2343	0.053*	
C18	−0.0310 (4)	0.2481 (3)	0.41507 (16)	0.0395 (6)	
H18	−0.1241	0.3154	0.3918	0.047*	
C19	−0.1320 (4)	−0.0336 (3)	0.26089 (15)	0.0378 (6)	
C20	−0.2003 (4)	−0.1679 (2)	0.25656 (14)	0.0339 (5)	
C21	0.2174 (4)	0.0674 (3)	0.39949 (17)	0.0431 (6)	
H21	0.3021	0.0059	0.3655	0.052*	
C22	−0.0351 (4)	0.2482 (3)	0.08150 (16)	0.0387 (6)	
H22	−0.1307	0.3146	0.1075	0.046*	
C23	−0.0687 (4)	−0.2874 (3)	0.25844 (15)	0.0354 (6)	
H23	0.0658	−0.2846	0.2642	0.042*	
C24	0.0186 (4)	−0.5397 (3)	0.25266 (14)	0.0372 (6)	

### Atomic displacement parameters (Å^2^)

**Table:**

	*U*^11^	*U*^22^	*U*^33^	*U*^12^	*U*^13^	*U*^23^
Cu1	0.0415 (2)	0.0285 (2)	0.0384 (2)	−0.00438 (14)	−0.00604 (14)	−0.00113 (13)
O1	0.0404 (11)	0.0330 (10)	0.0747 (14)	0.0028 (9)	−0.0121 (10)	−0.0069 (9)
O2	0.0470 (11)	0.0290 (9)	0.0466 (11)	−0.0040 (8)	−0.0094 (8)	−0.0024 (8)
O3	0.0392 (11)	0.0355 (11)	0.0691 (13)	−0.0008 (8)	−0.0099 (9)	−0.0049 (9)
O4	0.0375 (11)	0.0304 (9)	0.0458 (10)	−0.0047 (8)	−0.0059 (8)	−0.0023 (8)
O5	0.0368 (11)	0.0431 (11)	0.0589 (12)	0.0013 (9)	−0.0076 (9)	−0.0044 (10)
N1	0.0413 (13)	0.0499 (14)	0.0436 (13)	−0.0085 (11)	−0.0062 (10)	0.0074 (11)
N2	0.0458 (14)	0.0469 (14)	0.0449 (13)	−0.0048 (11)	−0.0044 (10)	−0.0075 (11)
N3	0.0350 (12)	0.0339 (12)	0.0426 (12)	−0.0071 (9)	−0.0040 (9)	−0.0024 (9)
N4	0.0352 (12)	0.0328 (11)	0.0418 (12)	−0.0066 (9)	−0.0056 (9)	0.0016 (9)
C1	0.0559 (19)	0.0586 (19)	0.0410 (15)	−0.0030 (15)	−0.0063 (13)	−0.0072 (13)
C2	0.0533 (19)	0.066 (2)	0.0407 (16)	−0.0018 (16)	−0.0055 (13)	0.0061 (14)
C3	0.0456 (17)	0.067 (2)	0.0419 (16)	−0.0055 (15)	−0.0038 (13)	−0.0053 (14)
C4	0.0495 (18)	0.0454 (16)	0.0527 (17)	−0.0073 (14)	−0.0024 (13)	−0.0080 (13)
C5	0.0420 (16)	0.0569 (19)	0.0507 (17)	−0.0013 (14)	−0.0009 (13)	−0.0115 (14)
C6	0.0517 (18)	0.0451 (16)	0.0506 (16)	−0.0115 (14)	−0.0112 (13)	0.0081 (13)
C7	0.0392 (15)	0.0537 (18)	0.0421 (15)	−0.0096 (13)	−0.0056 (12)	0.0015 (13)
C8	0.0461 (17)	0.0499 (17)	0.0462 (16)	−0.0089 (13)	−0.0024 (13)	−0.0101 (13)
C9	0.0476 (17)	0.0522 (18)	0.0477 (16)	−0.0113 (14)	−0.0086 (13)	0.0127 (13)
C10	0.0508 (18)	0.0531 (18)	0.0429 (15)	−0.0105 (14)	−0.0099 (13)	0.0021 (13)
C11	0.0417 (15)	0.0468 (16)	0.0413 (15)	−0.0095 (13)	−0.0055 (12)	−0.0004 (12)
C12	0.0352 (14)	0.0391 (15)	0.0431 (15)	0.0011 (11)	−0.0025 (11)	−0.0034 (11)
C13	0.0416 (16)	0.0442 (16)	0.0472 (16)	−0.0043 (12)	−0.0062 (12)	−0.0029 (12)
C14	0.0395 (14)	0.0354 (14)	0.0304 (12)	−0.0058 (11)	−0.0024 (10)	−0.0013 (10)
C15	0.0434 (16)	0.0361 (14)	0.0478 (15)	−0.0091 (12)	−0.0020 (12)	−0.0037 (12)
C16	0.0427 (16)	0.0483 (17)	0.0521 (17)	−0.0059 (13)	−0.0116 (13)	0.0102 (13)
C17	0.0337 (14)	0.0458 (16)	0.0531 (16)	−0.0079 (12)	−0.0056 (12)	−0.0040 (12)
C18	0.0405 (15)	0.0362 (14)	0.0412 (14)	−0.0047 (12)	−0.0045 (11)	−0.0001 (11)
C19	0.0414 (15)	0.0341 (14)	0.0376 (13)	−0.0021 (12)	−0.0095 (11)	−0.0004 (11)
C20	0.0380 (14)	0.0312 (13)	0.0321 (12)	−0.0039 (11)	−0.0030 (10)	−0.0009 (10)
C21	0.0402 (15)	0.0407 (15)	0.0476 (15)	−0.0033 (12)	−0.0045 (12)	−0.0018 (12)
C22	0.0395 (15)	0.0373 (14)	0.0395 (14)	−0.0068 (12)	−0.0041 (11)	−0.0015 (11)
C23	0.0350 (14)	0.0358 (14)	0.0352 (13)	−0.0035 (11)	−0.0050 (10)	−0.0023 (10)
C24	0.0448 (16)	0.0319 (14)	0.0338 (13)	−0.0031 (12)	−0.0028 (11)	−0.0012 (10)

### Geometric parameters (Å, °)

**Table:**

Cu1—O2^i^	1.9322 (18)	C5—H5	0.9500
Cu1—O4	1.9978 (18)	C6—C9^iii^	1.503 (4)
Cu1—N4	2.009 (2)	C6—H6A	0.9900
Cu1—N3	2.017 (2)	C6—H6B	0.9900
Cu1—O5	2.343 (2)	C7—C22	1.392 (4)
O1—C19	1.242 (3)	C8—C4^ii^	1.508 (4)
O2—C24	1.280 (3)	C8—H8A	0.9900
O3—C24	1.233 (3)	C8—H8B	0.9900
O4—C19	1.272 (3)	C9—C6^iii^	1.503 (4)
O5—H5A	0.820 (18)	C9—H9A	0.9900
O5—H5B	0.825 (17)	C9—H9B	0.9900
N1—C2	1.451 (4)	C10—C16	1.380 (4)
N1—C6	1.458 (4)	C10—C11	1.385 (4)
N1—C9	1.465 (3)	C10—H10	0.9500
N2—C1	1.452 (4)	C11—C18	1.389 (4)
N2—C8	1.461 (4)	C12—C17	1.384 (4)
N2—C4	1.462 (4)	C12—C20	1.401 (4)
N3—C13	1.337 (4)	C12—H12	0.9500
N3—C22	1.341 (3)	C13—H13	0.9500
N4—C18	1.338 (3)	C14—C15	1.389 (4)
N4—C21	1.342 (3)	C14—C23	1.390 (4)
C1—C11	1.506 (4)	C14—C24	1.511 (4)
C1—H1A	0.9900	C15—C17	1.386 (4)
C1—H1B	0.9900	C15—H15	0.9500
C2—C7	1.513 (4)	C16—C21	1.374 (4)
C2—H2A	0.9900	C16—H16	0.9500
C2—H2B	0.9900	C17—H17	0.9500
C3—C7	1.379 (4)	C18—H18	0.9500
C3—C5	1.381 (4)	C19—C20	1.505 (4)
C3—H3	0.9500	C20—C23	1.385 (4)
C4—C8^ii^	1.508 (4)	C21—H21	0.9500
C4—H4A	0.9900	C22—H22	0.9500
C4—H4B	0.9900	C23—H23	0.9500
C5—C13	1.380 (4)		
			
O2^i^—Cu1—O4	153.48 (8)	C22—C7—C2	122.2 (3)
O2^i^—Cu1—N4	93.47 (8)	N2—C8—C4^ii^	110.0 (2)
O4—Cu1—N4	89.57 (8)	N2—C8—H8A	109.7
O2^i^—Cu1—N3	91.23 (8)	C4^ii^—C8—H8A	109.7
O4—Cu1—N3	88.28 (8)	N2—C8—H8B	109.7
N4—Cu1—N3	173.41 (8)	C4^ii^—C8—H8B	109.7
O2^i^—Cu1—O5	95.11 (8)	H8A—C8—H8B	108.2
O4—Cu1—O5	111.24 (7)	N1—C9—C6^iii^	110.4 (2)
N4—Cu1—O5	90.05 (8)	N1—C9—H9A	109.6
N3—Cu1—O5	84.93 (8)	C6^iii^—C9—H9A	109.6
C24—O2—Cu1^iv^	130.85 (17)	N1—C9—H9B	109.6
C19—O4—Cu1	101.25 (15)	C6^iii^—C9—H9B	109.6
Cu1—O5—H5A	129 (2)	H9A—C9—H9B	108.1
Cu1—O5—H5B	95 (2)	C16—C10—C11	119.7 (3)
H5A—O5—H5B	116 (3)	C16—C10—H10	120.1
C2—N1—C6	112.3 (2)	C11—C10—H10	120.1
C2—N1—C9	110.0 (2)	C10—C11—C18	117.2 (3)
C6—N1—C9	108.5 (2)	C10—C11—C1	120.6 (3)
C1—N2—C8	111.6 (2)	C18—C11—C1	122.1 (2)
C1—N2—C4	112.1 (2)	C17—C12—C20	119.7 (2)
C8—N2—C4	109.2 (2)	C17—C12—H12	120.1
C13—N3—C22	118.3 (2)	C20—C12—H12	120.1
C13—N3—Cu1	118.38 (18)	N3—C13—C5	122.7 (3)
C22—N3—Cu1	123.13 (18)	N3—C13—H13	118.7
C18—N4—C21	117.8 (2)	C5—C13—H13	118.7
C18—N4—Cu1	122.48 (18)	C15—C14—C23	119.1 (2)
C21—N4—Cu1	119.72 (18)	C15—C14—C24	121.1 (2)
N2—C1—C11	113.3 (2)	C23—C14—C24	119.8 (2)
N2—C1—H1A	108.9	C17—C15—C14	120.2 (3)
C11—C1—H1A	108.9	C17—C15—H15	119.9
N2—C1—H1B	108.9	C14—C15—H15	119.9
C11—C1—H1B	108.9	C21—C16—C10	119.0 (3)
H1A—C1—H1B	107.7	C21—C16—H16	120.5
N1—C2—C7	114.5 (2)	C10—C16—H16	120.5
N1—C2—H2A	108.6	C12—C17—C15	120.6 (3)
C7—C2—H2A	108.6	C12—C17—H17	119.7
N1—C2—H2B	108.6	C15—C17—H17	119.7
C7—C2—H2B	108.6	N4—C18—C11	123.7 (2)
H2A—C2—H2B	107.6	N4—C18—H18	118.2
C7—C3—C5	120.2 (3)	C11—C18—H18	118.2
C7—C3—H3	119.9	O1—C19—O4	123.2 (3)
C5—C3—H3	119.9	O1—C19—C20	119.6 (2)
N2—C4—C8^ii^	109.8 (2)	O4—C19—C20	117.2 (2)
N2—C4—H4A	109.7	C23—C20—C12	119.1 (2)
C8^ii^—C4—H4A	109.7	C23—C20—C19	121.0 (2)
N2—C4—H4B	109.7	C12—C20—C19	119.9 (2)
C8^ii^—C4—H4B	109.7	N4—C21—C16	122.5 (3)
H4A—C4—H4B	108.2	N4—C21—H21	118.7
C13—C5—C3	118.4 (3)	C16—C21—H21	118.7
C13—C5—H5	120.8	N3—C22—C7	122.9 (3)
C3—C5—H5	120.8	N3—C22—H22	118.6
N1—C6—C9^iii^	110.4 (2)	C7—C22—H22	118.6
N1—C6—H6A	109.6	C20—C23—C14	121.3 (3)
C9^iii^—C6—H6A	109.6	C20—C23—H23	119.4
N1—C6—H6B	109.6	C14—C23—H23	119.4
C9^iii^—C6—H6B	109.6	O3—C24—O2	125.9 (2)
H6A—C6—H6B	108.1	O3—C24—C14	120.1 (2)
C3—C7—C22	117.6 (3)	O2—C24—C14	114.0 (2)
C3—C7—C2	120.2 (3)		
			
O2^i^—Cu1—O4—C19	−1.3 (3)	C22—N3—C13—C5	0.3 (4)
N4—Cu1—O4—C19	95.58 (16)	Cu1—N3—C13—C5	−174.6 (2)
N3—Cu1—O4—C19	−90.64 (16)	C3—C5—C13—N3	0.4 (4)
O5—Cu1—O4—C19	−174.53 (15)	C23—C14—C15—C17	−0.7 (4)
O2^i^—Cu1—N3—C13	156.3 (2)	C24—C14—C15—C17	−178.8 (2)
O4—Cu1—N3—C13	−50.2 (2)	C11—C10—C16—C21	−0.2 (4)
O5—Cu1—N3—C13	61.3 (2)	C20—C12—C17—C15	1.0 (4)
O2^i^—Cu1—N3—C22	−18.4 (2)	C14—C15—C17—C12	−0.2 (4)
O4—Cu1—N3—C22	135.1 (2)	C21—N4—C18—C11	−0.6 (4)
O5—Cu1—N3—C22	−113.4 (2)	Cu1—N4—C18—C11	178.0 (2)
O2^i^—Cu1—N4—C18	24.1 (2)	C10—C11—C18—N4	0.0 (4)
O4—Cu1—N4—C18	−129.5 (2)	C1—C11—C18—N4	178.2 (3)
O5—Cu1—N4—C18	119.3 (2)	Cu1—O4—C19—O1	−7.3 (3)
O2^i^—Cu1—N4—C21	−157.3 (2)	Cu1—O4—C19—C20	171.84 (18)
O4—Cu1—N4—C21	49.1 (2)	C17—C12—C20—C23	−1.0 (4)
O5—Cu1—N4—C21	−62.1 (2)	C17—C12—C20—C19	177.2 (2)
C8—N2—C1—C11	−161.2 (3)	O1—C19—C20—C23	−171.1 (2)
C4—N2—C1—C11	75.9 (3)	O4—C19—C20—C23	9.6 (4)
C6—N1—C2—C7	−72.1 (3)	O1—C19—C20—C12	10.7 (4)
C9—N1—C2—C7	166.9 (3)	O4—C19—C20—C12	−168.6 (2)
C1—N2—C4—C8^ii^	−176.4 (2)	C18—N4—C21—C16	0.8 (4)
C8—N2—C4—C8^ii^	59.3 (3)	Cu1—N4—C21—C16	−177.8 (2)
C7—C3—C5—C13	−0.4 (5)	C10—C16—C21—N4	−0.5 (4)
C2—N1—C6—C9^iii^	179.3 (2)	C13—N3—C22—C7	−1.1 (4)
C9—N1—C6—C9^iii^	−58.8 (3)	Cu1—N3—C22—C7	173.6 (2)
C5—C3—C7—C22	−0.3 (4)	C3—C7—C22—N3	1.1 (4)
C5—C3—C7—C2	177.2 (3)	C2—C7—C22—N3	−176.4 (3)
N1—C2—C7—C3	166.5 (3)	C12—C20—C23—C14	0.2 (4)
N1—C2—C7—C22	−16.1 (4)	C19—C20—C23—C14	−178.0 (2)
C1—N2—C8—C4^ii^	176.0 (2)	C15—C14—C23—C20	0.6 (4)
C4—N2—C8—C4^ii^	−59.4 (3)	C24—C14—C23—C20	178.8 (2)
C2—N1—C9—C6^iii^	−177.9 (3)	Cu1^iv^—O2—C24—O3	4.6 (4)
C6—N1—C9—C6^iii^	58.8 (3)	Cu1^iv^—O2—C24—C14	−174.75 (15)
C16—C10—C11—C18	0.4 (4)	C15—C14—C24—O3	−178.8 (2)
C16—C10—C11—C1	−177.8 (3)	C23—C14—C24—O3	3.1 (4)
N2—C1—C11—C10	−171.6 (3)	C15—C14—C24—O2	0.6 (4)
N2—C1—C11—C18	10.3 (4)	C23—C14—C24—O2	−177.5 (2)

Symmetry codes: (i) *x*, *y*+1, *z*; (ii) −*x*−1, −*y*+1, −*z*+1; (iii) −*x*−1, −*y*+1, −*z*; (iv) *x*, *y*−1, *z*.

### Hydrogen-bond geometry (Å, °)

**Table:**

*D*—H···*A*	*D*—H	H···*A*	*D*···*A*	*D*—H···*A*
O5—H5A···O1^v^	0.82 (2)	1.96 (2)	2.778 (3)	174 (3)
O5—H5B···O3^i^	0.83 (2)	2.02 (2)	2.805 (3)	158 (3)

Symmetry codes: (v) *x*+1, *y*, *z*; (i) *x*, *y*+1, *z*.
